# Late presentation of superior mesenteric artery syndrome following scoliosis surgery: a case report

**DOI:** 10.1186/1752-1947-2-9

**Published:** 2008-01-19

**Authors:** Athanasios I Tsirikos, Raymond E Anakwe, Alexander DL Baker

**Affiliations:** 1 Scottish National Spine Deformity Centre, Royal Hospital for Sick Children, Edinburgh, UK; 2 Consultant Orthopaedic and Spinal Surgeon, Honorary Clinical Senior Lecturer-University of Edinburgh, c/o Scottish National Spine Deformity Centre, Royal Hospital for Sick Children, Sciennes Road, Edinburgh, EH9 1LF, UK

## Abstract

**Introduction:**

Obstruction of the third part of the duodenum by the superior mesenteric artery (SMA) can occur following surgical correction of scoliosis. The condition most commonly occurs in significantly underweight patients with severe deformities during the first few days to a week following spinal surgery.

**Case presentation:**

We present the atypical case of a patient with normal body habitus and a 50° adolescent idiopathic thoracolumbar scoliosis who underwent anterior spinal arthrodesis with instrumentation and developed SMA syndrome due to progressive weight loss several weeks postoperatively. The condition manifested with recurrent vomiting, abdominal distension, marked dehydration, and severe electrolyte disorder. Prolonged nasogastric decompression and nasojejunal feeding resulted in resolution of the symptoms with no recurrence at follow-up. The spinal instrumentation was retained and a solid spinal fusion was achieved with good spinal balance in both the coronal and sagittal planes.

**Conclusion:**

SMA syndrome can occur much later than previously reported and with potentially life-threatening symptoms following scoliosis correction. Early recognition of the condition and institution of appropriate conservative measures is critical to prevent the development of severe complications including the risk of death.

## Introduction

**Figure 1:**
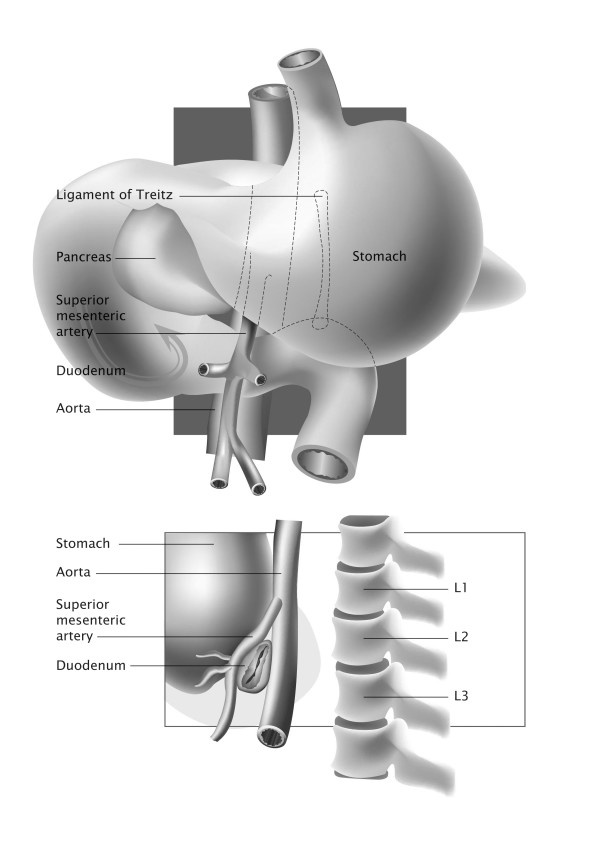
Graphical representation of the anatomical relations between the duodenum and the aortomesenteric angle.

Vascular compression of the third part of the duodenum by the SMA results in the development of a rare condition of gastric outlet occlusion known as SMA syndrome. The etiology of the syndrome is connected to the anatomy of the third part of the duodenum in relation to the aortomesenteric angle (Figure 1). Obstruction of the small bowel by the SMA has been previously associated with spinal manipulation in the surgical or conservative management of scoliosis and has also been described in cases of extensive burns, major surgeries such as ileoanal pouch anastomosis, multiple combat injuries, severe trauma, considerable weight loss in patients with malignancies, anorexia nervosa or other eating disorders, as well as following the application of spica casts [1-3].

In scoliosis, the syndrome occurs most commonly in thin and asthenic patients with a low body mass index (BMI) who undergo spinal manipulation and correction of the curvature by instrumentation, skeletal traction, casting or bracing; these corrective techniques all result in significant lengthening of the vertebral column and an extrinsic compression of the distal duodenum as it passes through the sharp angle formed by the aorta and the spine posteriorly and the SMA anteriorly. Following scoliosis surgery, the condition usually develops during the first postoperative week [4-6].

We present a patient with an adolescent idiopathic scoliosis who underwent anterior spinal arthrodesis and developed severe SMA syndrome 6.5 weeks following surgery. To the authors' knowledge this patient constitutes the latest presentation of SMA syndrome following spinal deformity surgery reported in the literature.

## Case presentation

A previously healthy 16.8-year-old caucasian girl presented to our institution with an adolescent idiopathic thoracolumbar scoliosis. Her past medical history was non-contributory in regard to operations, medication, or allergies. In admission, her body weight was 56.6 kg and her body height was 164 cm, which were both at the 50^th ^percentile for sex- and age-matched normal population. Her BMI was 21 kg/cm/cm, which was also at the 50^th ^percentile for her age and gender. There was no family history of scoliosis or gastrointestinal pathology.

**Figure 2:**
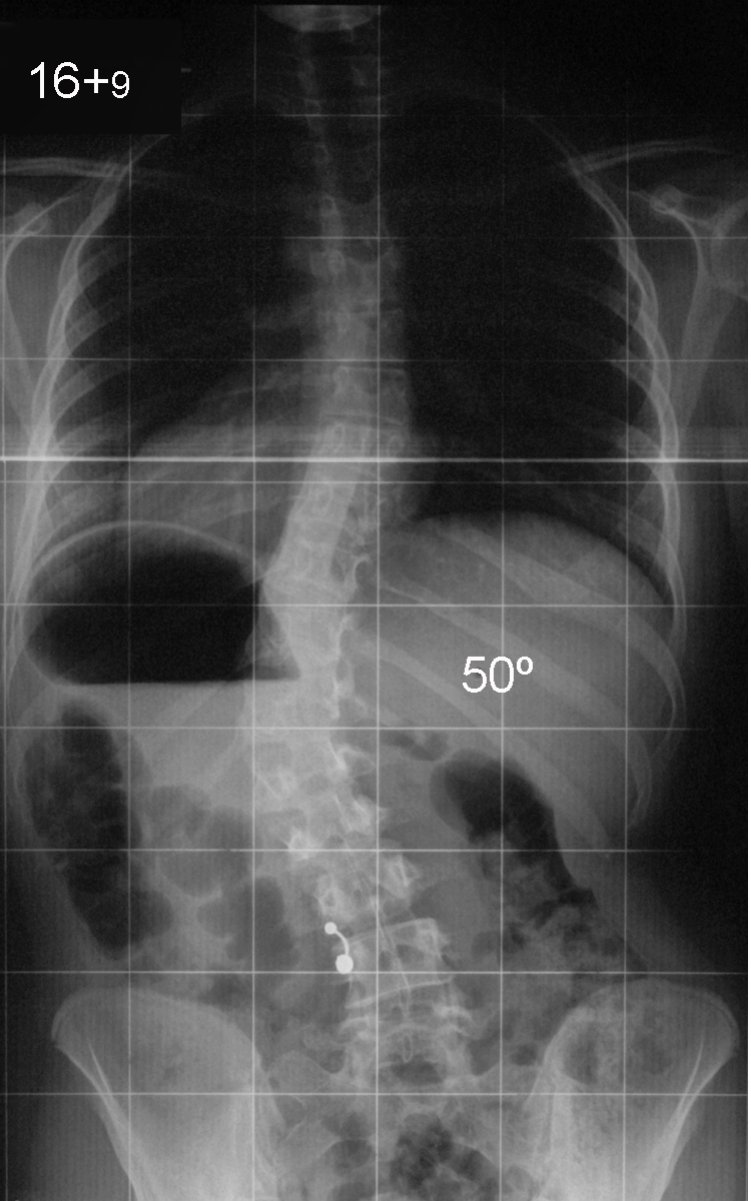
Preoperative anteroposterior radiograph of the spine shows a left thoracolumbar scoliosis extending from T10 to L2 and measuring 50°.

Our patient developed a flexible left thoracolumbar curve extending from T10 to L2 and measuring 50° (Figure 2). This was producing a moderate deformity due to listing of the trunk and thoracic translocation to the left and a significant waistline asymmetry. The preoperative blood screening revealed no pathological findings. Albumin, white blood cell, and lymphocyte counts were all within normal limits. She underwent an anterior spinal arthrodesis extending from T10 to L2 with the use of third generation instrumentation [AO-Universal Spine System (USS) II, Stratec Medical, Oberdorf] applying a spinal derotational effect and autologous rib bone graft performed through a left thoracoabdominal retroperitoneal approach to the spine. This achieved a very satisfactory correction of her scoliosis to 7° and a well-balanced spine in both the coronal and the sagittal planes. Intraoperative blood loss was 200 mls.

The patient's immediate postoperative course was uncomplicated and she was started on oral feedings at postoperative day 1. The chest drain was removed on postoperative day 3 and the patient was fitted with a thoracolumbar plaster jacket to provide additional support to the spine and mobilise out of her bed as per routine procedure in our institution. Eight days following surgery she had no complaints of her spine, was mobilising satisfactorily and had a body weight of 52 kg (20^th ^percentile). She was subsequently discharged and was prescribed dietary supplements high in calories to achieve gradual increase in her body weight to preoperative values. Follow-up in the out-patient clinic was arranged at 3 weeks post-discharge.

**Figure 3:**
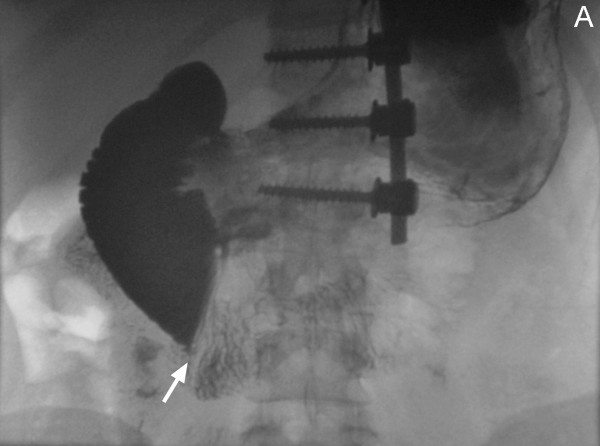
A barium contrast study shows dilatation of the stomach and the proximal duodenum, and occlusion of the third part of the duodenum by the SMA (white arrow).

The patient failed to attend her clinical appointment and was readmitted acutely at the hospital 45 days after spinal fusion due to the development of severe nausea and persistent vomiting. At the time of admission, she was markedly dehydrated with associated oliguria and severe electrolyte disorders including hypokalemia and metabolic alkalosis. Her body weight had dropped further to 45.2 kg, which was at the 3^rd ^percentile for gender- and age-matched normal population. This indicated a total loss of 11.4 kg post-surgery, which corresponded to 20% of her preoperative body weight. Her BMI was reduced to 16.8 kg/cm/cm, which was also below the 3^rd ^percentile for her age and gender. The spinal jacket was removed and on clinical examination her abdomen was found to be considerably distended but soft and non-tender, with normal bowel sounds. A barium contrast study was obtained and confirmed the clinical diagnosis of SMA syndrome (Figure 3).

A nasogastric (NG) tube was placed for drainage of the bilious gastric contents and a nasojejunal (NJ) tube under fluoroscopic guidance for feedings. Attention was drawn to correct electrolyte deficiencies through the administration of intravenous fluids. The nasogastric aspirates decreased gradually over the following 10 days. The patient received enteral feedings for a total period of 2 weeks. She was discharged 62 days post-surgery. Dietary supplements (calorific drinks) were prescribed for an additional period of 3 weeks. At the latest follow-up, 3.5 years after scoliosis correction, the patient was free of symptoms, had increased her body weight and BMI to the preoperative value of 50^th ^percentile of normal and her spine was fused without evidence of residual or recurrent deformity.

## Discussion

The incidence of SMA syndrome after surgical procedures to correct spinal deformities has been reported to vary between 0.5 and 4.7% [1,4,5,7-10]. The condition affects predominantly adolescent patients who are tall, slimly built with a thin asthenic body habitus. Children usually present for surgical correction of an adolescent idiopathic scoliosis during the phase of their most rapid longitudinal growth. This accelerated skeletal growth may alter the relation between the SMA and the spine by decreasing the aortomesenteric angle and, therefore, increase the risk for duodenal compression. The mechanism is that of an acute lengthening of the spinal column, which results in a cephalad displacement of the aorto-SMA junction at the expense of lateral mobility, due to either rapid height gain occurring during adolescence, or following correction of spinal deformities using either conservative (body casts and braces) or surgical methods.

The duodenum is surrounded by a mesenteric fat pad and lymphatic tissue as it crosses the aortomesenteric interval, which both serve as a cushion, allowing for sufficient space and preventing extrinsic obstruction of the bowel caused by the SMA. Any factors that obliterate this intervening space or disturb the relation between the adjacent anatomical structures may result in compression or occlusion of the duodenum. Underweight patients have less periduodenal fat to cushion and protect the duodenum in the SMA angle.

Surgical correction of the scoliotic spine produces vertical tension on the SMA and the mesentery and narrows even further the space available for the duodenum. In addition, the majority of patients with an adolescent idiopathic thoracic scoliosis have associated thoracic hypokyphosis, which by itself leads to a more extended spine and a reduced aortomesenteric angle. A developmentally short suspensory ligament of Treitz will also hold the duodenum in an elevated position, precipitating further constriction between the aorta and the SMA.

In a recent study, Braun et al. [9] reported on a group of patients who underwent scoliosis surgery and identified a staged procedure to the spine, a lumbar modifier of B or C as opposed to A, a low preoperative BMI, and increased stiffness of a thoracic scoliosis as the most predictive factors for the development of SMA syndrome.

The classic symptomatology of the condition includes nausea, bilious vomiting or increased bilious NG aspirates, postprandial abdominal fullness and distension, epigastric pain, while the bowel sounds are normal or hyperactive. The abdomen is soft with occasional tenderness in the epigastrium to deep palpation. The vomiting decompresses the stomach and produces asymptomatic intervals lasting several hours prior to the next episode.

The symptoms most commonly develop within a few days following scoliosis surgery and are associated with the use of both Harrington and third generation instrumentation techniques [4-6,11,12]. However, Kennedy and Cooper [13] reported a 14-year-old male patient who developed SMA syndrome and progressed rapidly to death 40 days after scoliosis correction with Harrington instrumentation and application of a body cast. Death in SMA syndrome can result from inhalation of vomitus or can be occasionally the consequence of gastric perforation [13]. In contrast, the patient described in the present study had a favourable outcome after conservative treatment, even though she developed the condition later than previously reported following an anterior spinal arthrodesis with third generation derotational instrumentation. The preoperative scoliotic curvature was not particularly severe and, therefore, our patient would not be regarded at high risk. The delay in the development of the syndrome can be attributed to the significant progressive weight loss that occurred in the postoperative period and resulted in gradual loss of the retroperitoneal fat in the aortomesenteric interval and subsequent obstruction of the duodenum.

The role of progressive postoperative weight loss has been increasingly identified as a critical factor predisposing in the occurrence of the condition [7,8]. This is in accordance with the current report of our patient who developed severe SMA syndrome resulting in significant medical illness 6.5 weeks following an anterior spinal arthrodesis. This patient was at the 50^th ^percentile for height, weight and BMI preoperatively for sex- and age-matched normal population. Both the weight and BMI progressively dropped to the 3^rd ^percentile following surgery. We, therefore, believe that the predominant etiological factor in the development of the SMA syndrome in this particular patient was the severe weight loss that occurred during the postoperative period. It is possible that the application of the spinal jacket could have caused extrinsic pressure to the abdomen, resulting in further decrease in the aortomesenteric angle and contributing to the onset of the symptoms. In addition, Crowther et al. [1] hypothesized that disruption of the autonomic nerve supply to the small intestine, which commonly occurs during the retroperitoneal dissection to approach anteriorly the thoracolumbar spine, can precipitate the development of the condition.

Initial treatment of SMA syndrome should involve conservative measures. The aim is to reverse the pathological cascade, which involves a primary partial duodenal obstruction secondary to anatomical features that progresses to complete occlusion through duodenal edema caused by the persistent vomiting and abdominal distension. Oral intake should be restricted. A NJ feeding tube should be passed distal to the site of the duodenal obstruction using radiographic assistance to provide enteral feedings and achieve gradual weight gain. It is important to note that gastric dilatation and recurrent vomiting can ultimately lead to progressive dehydration, severe hypovolemia, oliguria, electrolyte disorders, such as hypokalemia and metabolic alkalosis as occurred in our patient, or even death. Most of the reported deaths by the condition involve patients in whom the diagnosis was markedly delayed or was completely missed.

With appropriate conservative treatment, the symptoms usually regress after 2-3 days and oral intake with soft solids can be restarted under careful monitoring. If enteral feedings are not possible and the symptomatology persists, total parenteral nutrition should be introduced in order to provide adequate nutritional supplementation. Hospital stay is markedly protracted causing significant patient and parental anxiety. Surgical management should be considered only if conservative methods fail. Operative methods include open or laparoscopic duodenojejunostomy, division of the ligament of Treitz, and open derotation of the duodenum.

## Conclusion

We believe that it is essential to identify those patients who are at greater risk of developing duodenal obstruction and initiate intensive preoperative dietary supplementation in undernourished patients scheduled to undergo complex spine deformity surgery as a preventative measure. In addition, the significance of a close monitoring of any marked postoperative weight loss and the need for an early intervention cannot be overemphasised. We have described a patient who demonstrates that SMA syndrome can develop late following scoliosis surgery, a finding that is consistent with etiology of gradual onset. A high index of suspicion will lead to an early diagnosis of the condition at a stage when conservative measures are more likely to produce a good outcome. If the diagnosis is delayed or missed, SMA syndrome can cause considerable morbidity and may result in potentially life-threatening complications.

## Abbreviations

SMA: superior mesenteric artery

BMI: body mass index

NG: nasogastric

NJ: nasojejunal

## Competing interests

The author(s) declare that they have no competing interests.

## Authors' contributions

1). A.I. Tsirikos: conception and design, analysis of data, preparation of the manuscript, final approval of the version to be published.

2). R.E. Anakwe: acquisition and analysis of data, drafting the manuscript.

3). A.D.L. Baker: acquisition and analysis of data.

## Consent

Written informed consent was obtained from the patient for publication of the study. A copy of the written consent is available for review by the Editor-in-Chief of this journal.
